# Early introduction of baked egg (BE) in allergic patients in the first 2 years of life

**DOI:** 10.1186/2045-7022-5-S3-P38

**Published:** 2015-03-30

**Authors:** Angela Claver, Elena Botey, Begoña Navarro, Eladia Alarcón, Santiago Nevot, Anna Cisteró-Bahíma

**Affiliations:** 1 Servicio de Alergia, Hospital Universitari Quiron Dexeus, Barcelona, Spain

## Background

Previous studies suggest that extensive heating and food matrix diminish the allergenicity of egg white (EW) proteins, making it possible to be tolerated by some allergic patients.

## Methods

22 children were included: 18 with history of immediate reaction and 4 sensitized to egg (EW sIgE > 50 KU/L). All 22 underwent open OFC with BE performed in 3 days. Day one: OFC with cookies (brand containing egg). Tolerant patients incorporated cookies and food containing egg traces into their diet. A second challenge with home-breaded chicken was performed a week later. Regular consumption was advised for tolerant kids and 15 days later they were challenged with a serving size of home-made cake containing 3 eggs. All children continued regular ingestion and were periodically controlled. Factors including SPT and sIgE levels were used to determine a subsequent challenge with less-heated-egg.

## Results

All patients tolerated cookies. A 15 month old boy presented mild anaphylaxis (MA) during OFC with breaded chicken (exercise cofactor). Afterwards, he tolerated it and 1 year later passed the OFC with raw egg. A 14 month old girl presented MA during OFC with cake. She continued regular intake of breaded foods and cookies and tolerated omelet 12 months later. All the remaining patients tolerated BE without reactions. In the next 3-12 months 20/22 patients were successfully challenged with hard-boiled egg, 19/22 were challenged with omelet (16 passed/1 MA/ 2 cutaneous symptoms) and 3/22 with raw egg (2 passed/1 cutaneous symptoms). During home dosing only mild symptoms (perioral hives or sporadical vomiting) were observed in few patients.

## Comments

BE is well tolerated and safe. Thus, strict dietary avoidance may not be necessary, even from the diagnosis moment. Regular ingestion of HE could also change the natural course of egg allergy.

